# Subsequent high blood pressure and hypertension by hypertensive disorders of pregnancy: the Tohoku Medical Megabank Project Birth and Three-Generation Cohort Study

**DOI:** 10.1038/s41440-024-01936-9

**Published:** 2024-10-11

**Authors:** Mami Ishikuro, Taku Obara, Mayu Hasegawa, Keiko Murakami, Fumihiko Ueno, Aoi Noda, Tomomi Onuma, Fumiko Matsuzaki, Noriyuki Iwama, Masahiro Kikuya, Junichi Sugawara, Tatsuhiko Azegami, Takashin Nakayama, Asako Mito, Naoko Arata, Hirohito Metoki, Takeshi Kanda, Shinichi Kuriyama

**Affiliations:** 1 Department of Preventive Medicine and Epidemiology, Tohoku Medical Megabank Organization, Tohoku University, Sendai, Miyagi Japan; 2 Tohoku University Graduate School of Medicine, Sendai, Miyagi Japan; 3 Tohoku University Hospital, Sendai, Miyagi Japan; 4 Tohoku University School of Medicine, Sendai, Miyagi Japan; 5 Teikyo University School of Medicine, Itabashi-ku, Tokyo Japan; 6 Department of Internal Medicine, Keio University School of Medicine, Shinjyuku-ku, Tokyo Japan; 7 Division of Maternal Medicine, Center for Maternal-Fetal, Neonatal, and Reproductive Medicine, National Center for Child Health and Development, Setagaya, Japan; 8 Faculty of Medicine, Tohoku Medical and Pharmaceutical University, Sendai, Miyagi Japan; 9 Department of Nephrology, Faculty of Medicine, Shimane University, Izumo, Shimane Japan; 10 International Research Institute of Disaster Science, Tohoku University, Sendai, Miyagi Japan

**Keywords:** Blood pressure, Hypertension, Postpartum, Hypertensive disorders of pregnancy, Cohort study

## Abstract

Hypertensive disorders of pregnancy can cause hypertension in the future. Understanding how the blood pressure values of women with and without hypertensive disorders of pregnancy differ will facilitate follow-up blood pressure monitoring in clinical settings. This study investigated the association between hypertensive disorders of pregnancy and subsequent high blood pressure and hypertension. This study used Japanese data from the Tohoku Medical Megabank Project Birth and Three-Generation Cohort Study. Follow-up systolic and diastolic blood pressures in normotensive women during pregnancy and those with hypertensive disorders of pregnancy were estimated using analysis of covariance adjusted for women with low birthweight, history of gestational diabetes mellitus, age, body mass index, pulse rate, smoking and drinking at the follow-up assessment, paternal hypertension history, and maternal hypertension or hypertensive disorders of pregnancy history. Finally, we performed a multiple logistic regression analysis. In total, 7343 women were included in the analysis. Women with a history of hypertensive disorders of pregnancy had higher blood pressure approximately three years postpartum compared with normotensive women. Hypertensive disorders of pregnancy in the most recent pregnancy in different subgroups, such as nulliparous women, multiparous women without a history of hypertensive disorders of pregnancy, and multiparous women with a history of hypertensive disorders of pregnancy, were associated with an increased risk of subsequent hypertension. Women’s birthweight was also weakly associated with hypertension. Even one experience of hypertensive disorders of pregnancy may contribute to elevated blood pressure and hypertension approximately three years postpartum. In addition, women’s birthweights may have a weak relationship with increasing blood pressure.

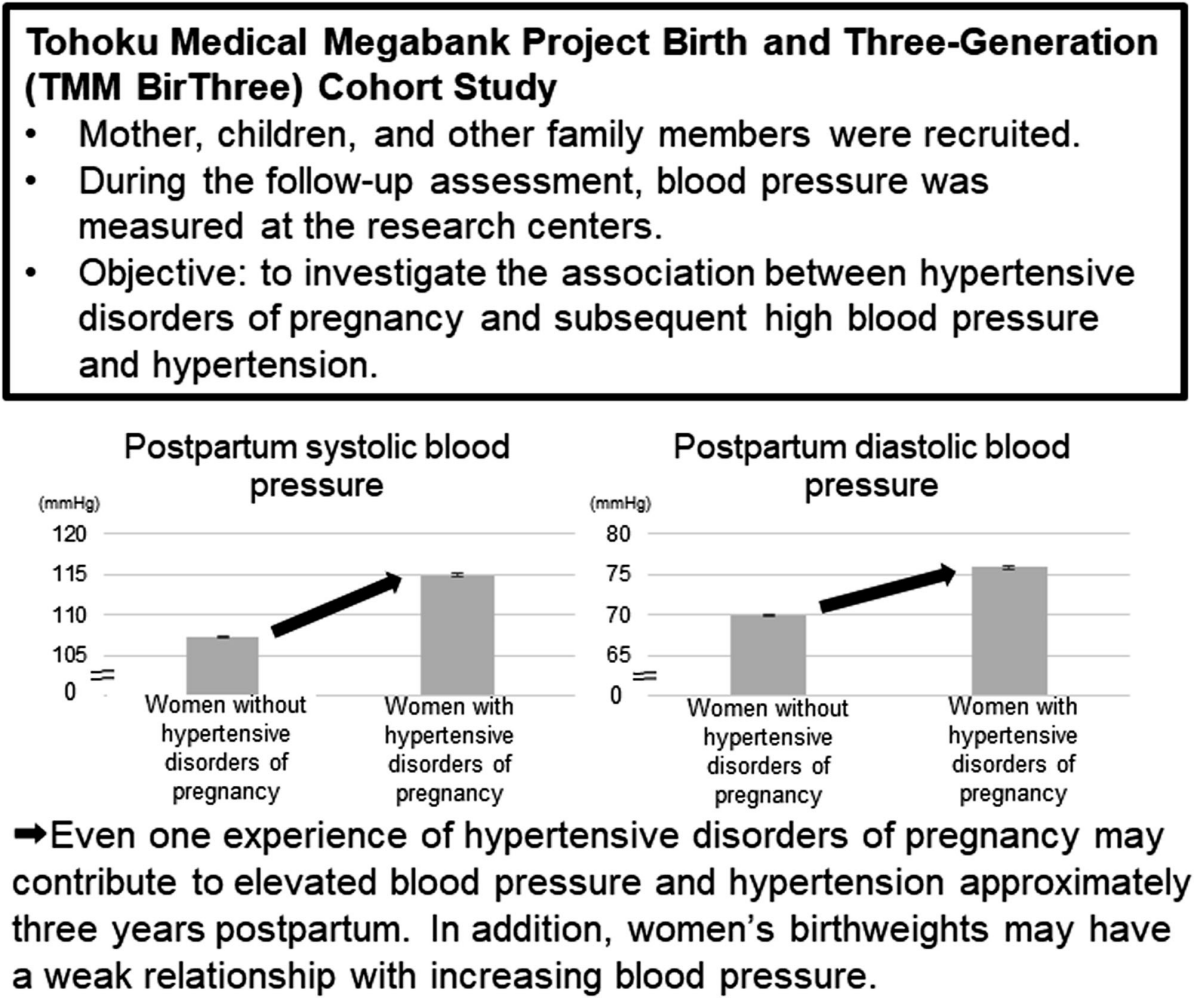

## Introduction

Hypertension is a common health concern worldwide. Approximately 1.39 billion people were estimated to have hypertension in 2010 [1]. In Japan, 43 out of 126 million people were estimated to have hypertension [2]. Moreover, hypertension or high blood pressure (BP) is an independent risk factor for future cardiovascular diseases [3]. Therefore, preventing or detecting high BP early to prevent more severe conditions, such as stroke, coronary heart disease, or death, is necessary [3].

Hypertensive disorders of pregnancy (HDP) cause chronic hypertension in women [4]. Mito et al. reported that hypertension incidence five years postpartum was higher in women who developed HDP than in normotensive women [5]. The Nurses’ Health Study II reported a long-term association between HDP and cardiovascular diseases [6]. HDP is a common obstetric disease that 5–10% of pregnant women develop worldwide [7, 8]. Therefore, postpartum BP monitoring is crucial. However, continuous postpartum monitoring has not been well established. Moreover, relatively younger women might have less opportunity to measure their BP frequently postpartum. Therefore, assessing whether HDP increases BP even a few years later postpartum is important. In a systematic review, HDP was found to be a risk factor for developing hypertension two years postpartum [9]. In addition, determining how the BP values of women with HDP differ from those of normotensive women will be important for underscoring the necessity of follow-up BP monitoring. Furthermore, determining the BP that should be particularly monitored will be beneficial in clinical settings to identify patients who require follow-up BP monitoring.

This study investigated the association between HDP and subsequent high BP and hypertension.

Point of view Clinical relevance: Even with one occurrence of HDP may increase the risk of future hypertension, and women’s birthweights may also have a weak relationship with increasing BP.

Future direction: Further studies should be conducted to determine whether women with low birthweight are a risk factor for their chronic hypertension.

Consideration for the Asian population: The prevalence of hypertension differs among countries and ethnicity. Home BP measurements especially in Japan might be useful and adaptable for BP monitoring.

## Methods

### Design and participants

The Tohoku Medical Megabank Organization (ToMMo)’s Internal Review Board approved this study (No. 2021-4-153). The data from the Tohoku Medical Megabank Project Birth and Three-Generation (TMM BirThree) Cohort Study was used for the analyses. The TMM BirThree Cohort Study investigated the effects of the disaster on people’s health, identified their needs for healthcare services, and established precise medicine/healthcare to better care for people living in the disaster region and throughout Japan. The ToMMo’s Internal Review Board approved the TMM BirThree Cohort Study’s protocol (no. 2013-1-103-1). Written informed consent for the TMM BirThree Cohort Study was obtained from all the participants. The detailed design of the study has been described previously [10, 11].

In the TMM BirThree Cohort Study, 23,406 pregnancies were registered. Participants who withdrew consent (607 pregnancies) and 904 pregnancies were excluded because some women participated in the TMM BirThree Cohort Study twice or more and the latest pregnancy information of women was used for the analysis. Participants who lacked essential information regarding HDP, HDP history, parity, and antihypertensive drug use during pregnancy, and those who missed BP assessment, were pregnant, and used antihypertensive drugs during the follow-up period were excluded (11,898 women). In addition, those with unknown covariates, such as women’s birthweight, history of gestational diabetes mellitus (GDM), age, body mass index (BMI), pulse rate, smoking, and drinking status during the follow-up assessment, and their parents’ history of hypertension (2654 women) were excluded (Supplementary Fig. 1).

### Measurements

HDP history prior to the TMM BirThree Cohort Study was obtained from a questionnaire that women filled out when they participated. After being part of the TMM BirThree Cohort Study, HDP was defined in accordance with the criteria of the American College of Obstetricians and Gynecologists [12], using BP from medical records, or those who used antihypertensive drugs. The genome medical research coordinators of ToMMo at Tohoku University transcribed the medical record. Characteristics during pregnancy such as parity, systolic, and diastolic BPs in the first trimester were obtained from medical records.

The TMM BirThree Cohort Study invited participants next 6 months to 7 years once after initial delivery to their own research centers and measured BP, pulse rate, and other physiological examinations. Genome medical research coordinators measured participants’ BP twice by HEM-9000AI electronic upper arm-cuff device (OMRON Corporation, Kyoto, Japan) with seated position 1–2 min resting. The mean BP values were used for the analysis. Regarding the logistic regression analysis, systolic BP ≥ 140 mmHg or diastolic BP ≥ 90 mmHg was defined as hypertension.

The women’s birthweights were obtained one year postpartum using a mailed questionnaire. Their answers were categorical variables as follows: <1500 g, ≥1500 g and <2000 g, ≥2000 g and < 2500 g, ≥2500 g and <3000 g, ≥3000 g and <3500 g, ≥3500 g and <4000 g, ≥4000 g, and unknown. Weights <2500 g were categorized as low after unknown and missing data were excluded. History of GDM was extracted from both medical records and questionnaires.

Age at the BP measurements was used for the analysis. The genome medical research coordinators determined their BMI via InBody 720 (Biospace Co Ltd., Seoul, Korea) using their height and categorized them as ≥ or <25 kg/m^2^. The pulse rate (per minute) was measured simultaneously with the BP using HEM-9000AI, and the mean of the pulse rate measured twice was used for the analysis. Smoking and drinking status were assessed using an electronic questionnaire at the research center. These variables were categorized as ever smoker/ever drinker and non-smoker/none drinker. Parents’ hypertension history, including the maternal HDP history, was obtained from questionnaires participants filled out and brought when they visited our research centers.

### Statistical analysis

The proportion of women with low birthweight, history of GDM, ever smoker/drinker, and parents’ hypertension history were compared between normotensive women and those with HDP during pregnancy using the chi-squared test. Age, BMI, and pulse rate at follow-up assessment were compared using a *t*-test. Years from delivery to follow-up assessment were also compared using the *t*-test.

Systolic and diastolic BP at the follow-up assessment in normotensive women and those with HDP history were estimated and compared using analysis of covariance adjusted for women with low birthweight, history of GDM, age, BMI, pulse rate, smoking and drinking at the follow-up assessment, paternal hypertension history, and maternal hypertension or HDP history.

Multiple logistic regression analysis assessed the association between HDP history and hypertension at the follow-up assessment. The logistic model was adjusted for women with low birthweight, history of GDM, age, BMI, pulse rate, smoking and drinking at the follow-up assessment, paternal hypertension history, and maternal hypertension or HDP history.

Sub-analyses were also conducted among study participants stratified into three groups: nulliparous women who delivered their children for the first time in this analysis, multiparous without HDP history in the delivery before the analysis, and multiparous women with HDP history in the delivery before the analysis. We investigated the association between HDP prevalence in the most recent pregnancy, indicating HDP prevalence in the BirThree Cohort Study, and subsequent BP level and hypertension. The characteristics were compared between normotensive women and those with HDP in the most recent pregnancy in each group using the chi-square test, Fisher’s exact test, or *t*-test. Follow-up systolic and diastolic BPs in normotensive women and those with HDP during the most recent pregnancy in each group were estimated using analysis of covariance adjusted for women with low birthweight, history of GDM, age, BMI, pulse rate, smoking, and drinking at the follow-up assessment, paternal hypertension history, and maternal hypertension or HDP history. Multiple logistic regression analysis assessed the association of HDP in the most recent pregnancy with hypertension at the follow-up assessment. The models were adjusted for women with low birthweight, history of GDM, age, BMI, pulse rate, smoking and drinking at the follow-up assessment, paternal hypertension history, and maternal hypertension or HDP history.

Multiple logistic regression analysis of women with low birthweight, parity, and HDP history was also performed. All statistical analyses were performed using SAS (version 9.4; SAS Institute Inc., Cary, NC, USA).

## Results

In total, 7343 women were included in the analysis. Women with HDP history accounted for 12.4%. At the follow-up assessment, the mean age of women with HDP history was 36.2 years, and that of normotensive women was 35.5 years (Table 1). BMI was higher in women with HDP history than in normotensive women. The proportion of smokers and years from the age at delivery to the follow-up assessment did not differ between women with HDP history and normotensive women. The proportion of women with a history of HDP who also had a history of parental hypertension was higher than that of normotensive women.

**Table 1:** Characteristics of normotensive women and those with HDP

	Normotensive women		Women with HDP		*P*-value
	(*n* = 6430)		(*n* = 913)		
Women with low birthweight, *n*, %	470	7.3	97	10.6	0.0004
GDM history and GDM during the most recent pregnancy, *n*, %	168	2.6	46	5.0	<0.0001
Age at the follow-up assessment (years)	35.5	4.8	36.2	5.1	<0.0001
BMI at the follow-up assessment (kg/m^2^)	21.6	3.4	23.6	4.9	<0.0001
Smoking at the follow-up assessment, *n*, %	449	7.0	78	8.5	0.09
Drinking at the follow-up assessment, *n*, %	2630	40.9	347	38.0	0.1
Paternal history of chronic hypertension, *n*, %	1651	25.7	288	31.5	0.0002
Maternal history of chronic hypertension or HDP, *n*, %	1383	21.5	260	28.5	<0.0001
Years from delivery to the follow-up assessment (years)	3.5	0.9	3.5	0.9	0.6
SBP at the follow-up assessment (mmHg)	107.0	10.2	117.2	15.3	<0.0001
DBP at the follow-up assessment (mmHg)	69.7	8.4	77.7	12.0	<0.0001
Hemoglobin A1c at the follow-up assessment (%)	5.3	0.3	5.4	0.4	<0.0001
Hypertension at the follow-up assessment, *n*, %	138	2.2	150	16.4	<0.0001

Continuous variables are presented as mean and standard deviation and categorical variables are presented as numbers and percentages. Hemoglobin A1c level of 6419 women with normotension and 911 women with HDP was measured

*HDP* hypertensive disorders of pregnancy, *BMI* body mass index, *GDM* gestational diabetes mellitus, *SBP* systolic blood pressure, *DBP* diastolic blood pressure

The mean and standard deviation of systolic and diastolic BPs at approximately three years were 117.2 ± 15.3/77.7 ± 12.0 mmHg in women with HDP and 107.0 ± 10.2/69.7 ± 8.4 mmHg in normotensive women (Table 1). The estimated mean and standard error of systolic and diastolic BPs adjusted for covariates were 114.9 ± 0.3/75.8 ± 0.3 mmHg in women with HDP and 107.3 ± 0.1/69.9 ± 0.1 mmHg in normotensive women (*P* < 0.0001).

Furthermore, 150 of 913 women with HDP history, and 138 of 6430 normotensive women had hypertension during the follow-up assessment (Table 1). Women with HDP history had a higher odds ratio (OR) than normotensive women (OR [95% CI] = 5.59 [4.29–7.29]) (Table 2).

**Table 2:** Factors associated with hypertension in approximately three years postpartum

	(*n* = 7343)	
	Adjusted OR	95%CI
Women with low birthweight	1.62	1.08–2.45
GDM history and GDM during the most recent pregnancy	1.01	0.56–1.82
Age at the follow-up assessment (years)	1.07	1.04–1.10
BMI at the follow-up assessment (kg/m^2^)	1.16	1.13–1.19
Pulse rate at the follow-up assessment (/min)	1.06	1.05–1.08
Smoking at the follow-up assessment	0.95	0.59–1.54
Drinking the follow-up assessment	1.47	1.13–1.91
Paternal history of chronic hypertension	1.60	1.22–2.10
Maternal history of chronic hypertension or HDP	2.05	1.57–2.69
HDP history and HDP during the most recent pregnancy	5.59	4.29–7.29

*HDP* hypertensive disorders of pregnancy, *BMI* body mass index, *GDM* gestational diabetes mellitus, *OR* odds ratio, *CI* confidence interval

Regarding the sub-analyses, 3066 nulliparous women, 4043 multiparous women without HDP history, and 234 multiparous women with HDP history were included. The proportion of HDP during the most recent pregnancy in each group was 12.3%, 7.5%, and 26.9% in nulliparous, multiparous women without HDP, and multiparous women with HDP, respectively. In each group, BMI and pulse rate were higher in women with HDP than in normotensive women during the most recent pregnancy at the follow-up assessment (Supplementary Table 1).

The means and standard deviations of systolic and diastolic BPs were 117.1 ± 15.2/77.8 ± 12.3 mmHg in women with HDP and 106.2 ± 9.8/69.3 ± 8.2 mmHg in normotensive women during the most recent pregnancy among nulliparous women. The means and standard deviations of systolic and diastolic BPs were 117.0 ± 15.1/77.8 ± 11.6 mmHg in women with HDP and 107.6 ± 10.4/69.9 ± 8.6 mmHg in normotensive women during the most recent pregnancy among multiparous women without HDP history. Similarly, among multiparous women with a history of HDP, the means and standard deviations of systolic and diastolic BPs were 131.6 ± 16.6/87.6 ± 13.9 mmHg for those with HDP, and 112.6 ± 11.8/73.8 ± 9.1 mmHg for normotensive women during their most recent pregnancy. Estimated means and standard errors of systolic and diastolic BPs adjusted for covariates in each group were; 114.4 ± 0.5/75.6 ± 0.4 mmHg in women with HDP and 106.6 ± 0.2/69.6 ± 0.2 mmHg in normotensive women during the most recent pregnancy (*P* < 0.0001) among nulliparous women; 115.2 ± 0.6/76.4 ± 0.5 mmHg in women with HDP and 107.7 ± 0.2/70.1 ± 0.1 mmHg in normotensive women during the most recent pregnancy (*P* < 0.0001) in multiparous women without HDP history; 128.5 ± 1.7/84.6 ± 1.4 mmHg in women with HDP and 113.7 ± 1.0/75.0 ± 0.8 mmHg in normotensive women in the most recent pregnancy (*P* < 0.0001) in multiparous women with HDP history (Supplementary Table 2).

The proportion of women with hypertension at the follow-up assessment among women with HDP was higher than that among normotensive women during the most recent pregnancy in each group (Supplementary Table 2). In multiparous women with HDP history, 42.9% of women with HDP during the most recent pregnancy had hypertension at the follow-up assessment. Women with HDP had higher OR for hypertension than normotensive women during the most recent pregnancy in each group (nulliparous women: OR [95% CI] = 5.59 [3.61–8.68], multiparous women without HDP history; OR [95% CI] = 5.67 [3.76–8.55], multiparous women with HDP history; OR [95% CI] = 3.24 [1.41–7.44]) (Supplementary Table 3). Women with low birthweight were associated with hypertension at the follow-up assessment in multiparous women with HDP history.

**Fig. 1:**
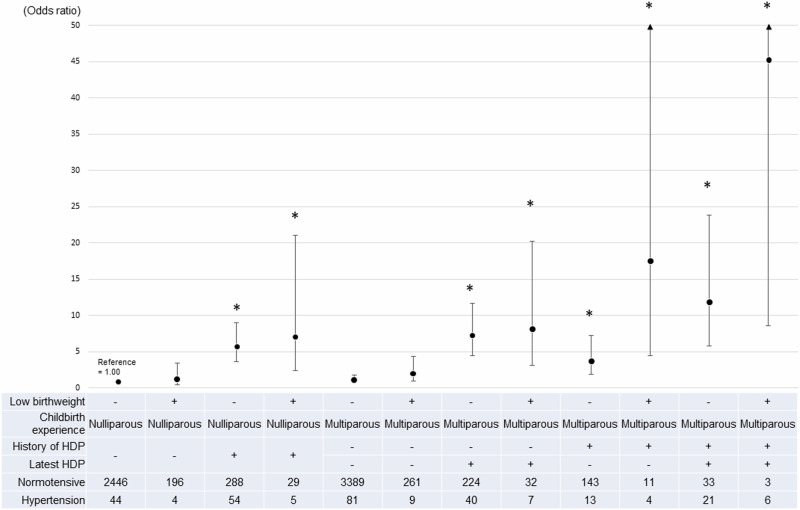
Comparison among women by birthweight, parity, HDP history, and HDP in the most recent pregnancy. Study participants were divided into 12 groups; (1) Low birthweight (−), nulliparous women latest HDP (−) (reference), (2) Low birthweight (+), nulliparous women latest HDP (−), (3) Low birthweight (−), nulliparous women latest HDP (+), (4) Low birthweight (+), nulliparous women latest HDP (+), (5) Low birthweight (−), multiparous women without HDP history, latest HDP (−), (6) Low birthweight (+), multiparous women without HDP history, latest HDP (−), (7) Low birthweight (−), multiparous women without HDP history, latest HDP (+), (8) Low birthweight (+), multiparous women without HDP history, latest HDP (+), (9) Low birthweight (−), multiparous women with HDP history, latest HDP (−), (10) Low birthweight (+), multiparous women with HDP history, latest HDP (−), (11) Low birthweight (−), multiparous women with HDP history, latest HDP (+), (12) Low birthweight (+), multiparous women with HDP history, latest HDP (+). Multiple logistic regression analysis adjusted for age, BMI, smoking and drinking status at the follow-up assessment, paternal hypertension history, maternal history of hypertension or HDP, systolic and diastolic BPs in the first trimester, and pulse rate was performed

Figure 1 presents the 12 categories based on women with low birthweight, parity, and HDP history. HDP history was strongly associated with hypertension during follow-up. In contrast, women with low birthweight were weakly associated with hypertension at follow-up.

## Discussion

This study demonstrated that women with HDP histories developed higher BP at approximately three years postpartum than those without HDP. HDP was also associated with an increased risk of hypertension three years postpartum. The study revealed that a single history of HDP could be a risk factor for subsequently developing high BP and hypertension. Furthermore, women’s birthweight was moderately associated with hypertension.

A previous meta-analysis and systematic review uncovered that HDP is a risk factor for hypertension within two years postpartum [9]. Studies have also reported the association between HDP and subsequent hypertension in ≥five years [5, 6]. The results of the present study are consistent with those of previous studies. Subsequent hypertension in multiparous women with HDP history was more than that in nulliparous women and multiparous women without HDP history. Nonetheless, the OR of HDP during the most recent pregnancy was lower than that in the other groups. Pregnant women with HDP history might have received intervention during the current pregnancy, preventing HDP reoccurrence. It is possible that such association could not be clearly assessed because this is an observational study. However, the association still existed, suggesting that HDP history can be a factor for the increased risk of subsequent hypertension. Moreover, studies have reported that HDP can be a risk factor for subsequent hypertension and cardiovascular or cerebrovascular diseases [13–17]. Leon et al. analyzed a large population of the United Kingdom with electronic health records. It was found that HDP history had higher hazard ratios for all stroke and cardiac atherosclerotic disease types [17]. Leon et al. also observed that the cumulative incidence of any cardiovascular disease among preeclamptic women was double that of normotensive women two years postpartum. Regarding screening for hypertension and preventing cardiovascular disease, the BP of women with HDP history should be actively and continuously monitored postpartum. However, there is no clear consensus on screening for the risk of postpartum hypertension or cardiovascular disease among various guidelines [18]. Japanese Society for the Study of Hypertension in Pregnancy recommends that women with HDP history undergo health check-ups once yearly [19]. Nevertheless, women taking maternity leave and women who do not work have fewer opportunities to take health check-ups. We observed subsequent hypertension and high BP. Therefore, monitoring BP at home might be effective. Home BP monitoring can reflect target end-organ damage more than monitoring BP at the office [20]. Hence, many countries recommend home BP monitoring [21–24]. In Japan, more than 15 million home BP measurement devices are manufactured every year, and 35 million units have been distributed to households [21]. Therefore, it might be useful for monitoring BP postpartum. In fact, a postpartum home BP monitoring program has been initiated in the United States [25]. Women who develop HDP should be informed of the risk of subsequent hypertension and asked to see a physician immediately when they have consistently high BP. According to a meta-analysis, diet and physical activity education might be effective in changing women’s lifestyles, although evidence regarding effective interventions that can reduce the risk of cardiovascular disease is lacking [26]. In addition to monitoring BP for the early detection of subsequent hypertension, intervention methods have to be established.

The mechanism by which HDP affects future chronic hypertension remains unclear; however, previous studies have suggested a shared background between HDP and subsequent hypertension. Placental ischemia caused by immune dysregulation causes endothelial dysfunction, increasing antiangiogenic proteins, such as soluble fms-like tyrosine kinase 1 (sFlt-1) and soluble endoglin. These antiangiogenic proteins accelerate endothelial dysfunction [27, 28]. Postpartum sFlt-1 usually decreases, however, remains higher in women with preeclampsia than in those who are normotensive during pregnancy [28]. Chambers reported that the mean brachial artery flow-mediated dilatation was lower in women with preeclampsia than in those who were normotensive at a median of three years postpartum [29], supporting the possibility of continuous endothelial dysfunction and this study’s results. Genetic HDP factors have also been reported in fetal and maternal genomes. Steinthorsdottir et al. recently identified [30] that fat mass and the obesity-associated (*FTO*) gene and a variant near *ZNF831* in maternal genomes which have been reported to be associated with BP [31–33] were also associated with preeclampsia. This study observed a relationship with hypertension similar to other studies [34, 35]. Genetic factors might partially explain this result. Furthermore, Stuart et al. mentioned that women with preeclampsia had slightly higher BP pre-pregnancy, indicating a pre-existing factor for future hypertension [6]. In contrast, Männistö et al. reported that HDP without known risk factors was associated with subsequent hypertension, indicating its independent relation to subsequent hypertension [36]. HDP could trigger and reflect hypertensive progression in the future.

Child-low birthweight is a possible factor for cardiovascular disease in women [37, 38]. Moreover, this study observed that women with low birthweight might be related to subsequent hypertension. Wagata et al. uncovered an association between women with low birthweight and HDP [39] and between HDP and subsequent hypertension in a different cohort study [40]. This study revealed a possible relationship between women with low birthweight and HDP on the risk for subsequent hypertension. Low birthweight is a risk factor for preeclampsia and chronic kidney disease, of which the latter is related to chronic hypertension [41]. Low birthweight might be a surrogate marker of lesser nephrons [42]. Further studies should be conducted to determine whether women with low birthweight are a risk factor for maternal chronic hypertension.

### Perspectives in Asia

While the prevalence of hypertension differs among countries and ethnicities, hypertension is a global concern. Shida et al. pointed that women and clinicians often discontinue BP surveillance as the BP levels return to normal within the 12 weeks postpartum; however, because of the subsequent risk, adequate follow-up after the BP levels return to normal is necessary [43]. Home BP measurement is one of the valuable methods to monitor BP [44, 45]. Although home BP measurement is not widely implemented in Asia, Japan promotes home BP measurement and the proportion of patients who have a device at home is high among Asian countries [46]. BP measurements at home might be also useful for Japanese women especially who have a history of HDP to detect signs of future hypertension.

### Strength and limitations

The strength of this study is that the data were from a large sample size using a standardized method. However, this study had some limitations. First, we could not follow all women three years postpartum, and we could not identify the onset of hypertension even among women who measured their BP at the follow-up assessment. Participants who underwent follow-up assessments and completed questionnaires might care more about their health, which could also make our analysis underestimate BP levels. Second, HDP history, parental hypertension history, and maternal birthweight data were obtained via questionnaires, which may have resulted in recall bias. Third, we did not measure BP more than twice; therefore, some women might have been overestimated as having hypertension. Finally, we did not consider HDP developed postpartum. We could not further investigate the remission status. Moreover, we could not limit the women with HDP to only those with a new onset of hypertension during pregnancy. Women with chronic hypertension might have been already managed by cardiologists; thus, it is not necessary to facilitate their follow-up. However, we still observed higher BP and a higher proportion of subsequent hypertension among women with a HDP history. Remarkably, we excluded the women who received antihypertensive drugs during the follow-up; nonetheless, 3.9% of all women in our study had hypertension. A history of HDP, even with one occurrence, may increase the risk of future hypertension.

## Conclusion

HDP may contribute to elevated BP approximately three years postpartum.

## Supplementary information

Supplementary Tables

Supplementary Figure 1

Supplementary Figure Legend
