# Supplementary material for: Subsequent high blood pressure and hypertension by hypertensive disorders of pregnancy: the Tohoku Medical Megabank Project Birth and Three-Generation Cohort Study
Source: Hypertens Res. 2024 Oct 11;48(1):68–76. doi: 10.1038/s41440-024-01936-9 (PMC11700841; doi:10.1038/s41440-024-01936-9)
Supplement: Supplementary file 1 — Supplementary Tables [file 41440_2024_1936_MOESM1_ESM.docx]

| Supplementary Table 1. Characteristics of women by parity and history of HDP before participating in the BirThree Cohort Study | | | | | |  |  |
| --- | --- | --- | --- | --- | --- | --- | --- |
|  | Normotensive women during the most recent pregnancy | | Women with HDP during the most recent pregnancy | | P-value |  |  |
| Nulliparous women | (n = 2,690) | | (n = 376) | |  |  |  |
| Women with low birthweight, n, % | 200 | 7.4 | 34 | 9.0 | 0.3 |  |  |
| GDM during the most recent pregnancy, n, % | 48 | 1.8 | 17 | 4.5 | 0.0006 |  |  |
| Age at the follow-up assessment (years) | 34.7 | 5.1 | 35.7 | 5.3 | 0.001 |  |  |
| BMI at the follow-up assessment (kg/m^2^) | 21.5 | 3.4 | 23.8 | 5.0 | <0.0001 |  |  |
| Pulse rate at the follow-up assessment (/min) | 61.4 | 7.6 | 64.3 | 9.5 | <0.0001 |  |  |
| Smoking at the follow-up assessment, n, % | 148 | 5.5 | 22 | 5.9 | 0.8 |  |  |
| Drinking at the follow-up assessment, n, % | 907 | 33.7 | 117 | 31.1 | 0.3 |  |  |
| Years from delivery to the follow-up assessment (years) | 3.6 | 0.8 | 3.6 | 0.8 | 0.1 |  |  |
| Paternal history of chronic hypertension, n, % | 727 | 27.0 | 114 | 30.3 | 0.2 |  |  |
| Maternal history of chronic hypertension or HDP, n, % | 621 | 23.1 | 115 | 30.6 | 0.001 |  |  |
| Hemoglobin A1c at the follow-up assessment (%) | 5.3 | 0.3 | 5.4 | 0.5 | <0.0001 |  |  |
| Multiparous women without HDP history | (n = 3,740) | | (n = 303) | |  |  |  |
| Women with low birthweight, n, % | 270 | 7.2 | 39 | 12.9 | 0.0004 |  |  |
| GDM history and GDM during the most recent pregnancy, n, % | 120 | 3.2 | 11 | 3.6 | 0.7 |  |  |
| Age at the follow-up assessment (years) | 36.0 | 4.5 | 36.2 | 4.9 | 0.6 |  |  |
| BMI at the follow-up assessment (kg/m^2^) | 21.6 | 3.5 | 23.1 | 4.6 | <0.0001 |  |  |
| Pulse rate at the follow-up assessment (/min) | 61.2 | 7.7 | 62.9 | 8.1 | 0.0003 |  |  |
| Smoking at the follow-up assessment, n, % | 301 | 8.1 | 31 | 10.2 | 0.2 |  |  |
| Drinking at the follow-up assessment, n, % | 1,723 | 46.1 | 135 | 44.6 | 0.6 |  |  |
| Years from delivery to the follow-up assessment (years) | 3.4 | 0.9 | 3.3 | 0.9 | 0.2 |  |  |
| Paternal history of chronic hypertension, n, % | 924 | 24.7 | 85 | 28.1 | 0.2 |  |  |
| Maternal history of chronic hypertension or HDP, n, % | 762 | 20.4 | 73 | 24.1 | 0.1 |  |  |
| Hemoglobin A1c at the follow-up assessment (%) | 5.3 | 0.3 | 5.4 | 0.3 | 0.1 |  |  |
| Multiparous women with HDP history | (n = 171) | | (n = 63) | |  |  |  |
| Women with low birthweight, n, % | 15 | 8.8 | 9 | 14.3 | 0.2 |  |  |
| GDM history and GDM during the most recent pregnancy, n, % | 14 | 8.2 | 4 | 6.4 | 0.8 |  |  |
| Age at the follow-up assessment (years) | 37.0 | 4.6 | 37.9 | 5.0 | 0.2 |  |  |
| BMI at the follow-up assessment (kg/m^2^) | 22.8 | 4.1 | 26.7 | 6.3 | <0.0001 |  |  |
| Pulse rate at the follow-up assessment (/min) | 61.2 | 7.0 | 66.7 | 9.9 | <0.0001 |  |  |
| Smoking at the follow-up assessment, n, % | 19 | 11.1 | 6 | 9.5 | 0.7 |  |  |
| Drinking at the follow-up assessment, n, % | 67 | 39.2 | 28 | 44.4 | 0.5 |  |  |
| Years from delivery to the follow-up assessment (years) | 3.6 | 1.0 | 3.5 | 1.1 | 0.8 |  |  |
| Paternal history of chronic hypertension, n, % | 56 | 32.8 | 33 | 52.4 | 0.006 |  |  |
| Maternal history of chronic hypertension or HDP, n, % | 51 | 29.8 | 21 | 33.3 | 0.6 |  |  |
| Hemoglobin A1c at the follow-up assessment (%) | 5.4 | 0.3 | 5.5 | 0.4 | 0.03 |  |  |
| HDP; hypertensive disorders of pregnancy, BMI; body mass index, GDM; gestational diabetes mellitus. | | | | | | | |
| Continuous variables are presented as mean and standard deviation, and categorical variables are presented as numbers and percentages.  Hemoglobin A1c level of 2,686 women with normotension and 375 women with HDP among nulliparous women, 3,733 women with normotension and 302 women with HDP among multiparous women without HDP history, and 171 women with normotension and 63 women with HDP among multiparous women without HDP history was measured. | | | | | | | |

| Supplementary Table 2. Blood pressure and proportion of hypertension in approximately three years postpartum between normotensive women and those with HDP in 3 groups by parity | | | | | |
| --- | --- | --- | --- | --- | --- |
|  |  |  |  |  |  |
|  |  |  |  |  |  |
|  | Normotensive women during the most recent pregnancy | | Women with HDP during the most recent pregnancy | | P-value |
| Nulliparous women | (n = 2,690) | | (n = 376) | |  |
| SBP (mmHg) | 106.6 | 0.2 | 114.4 | 0.5 | <0.0001 |
| DBP (mmHg) | 69.6 | 0.2 | 75.6 | 0.4 | <0.0001 |
| Hypertension, n, % | 48 | 1.8 | 59 | 15.7 | <0.0001 |
| Multiparous women without HDP history | (n = 3,740) | | (n = 303) | |  |
| SBP (mmHg) | 107.7 | 0.2 | 115.2 | 0.6 | <0.0001 |
| DBP (mmHg) | 70.1 | 0.1 | 76.4 | 0.5 | <0.0001 |
| Hypertension, n, % | 90 | 2.4 | 47 | 15.5 | <0.0001 |
| Multiparous women with HDP history | (n = 171) | | (n = 63) | |  |
| SBP (mmHg) | 113.7 | 1.0 | 128.5 | 1.7 | <0.0001 |
| DBP (mmHg) | 75.0 | 0.8 | 84.6 | 1.4 | <0.0001 |
| Hypertension, n, % | 17 | 9.9 | 27 | 42.9 | <0.0001 |
| HDP; hypertensive disorders of pregnancy, SBP; systolic blood pressure, DBP; diastolic blood pressure. | | | |  |  |
| All analyses were adjusted for covariates in each group. | |  |  |  |  |

| Supplementary Table 3. An association of HDP during the most recent pregnancy with hypertension in approximately three years postpartum | | | | | | | | | | | | | | |  |
| --- | --- | --- | --- | --- | --- | --- | --- | --- | --- | --- | --- | --- | --- | --- | --- |
|  |  |  |  |  |  |  |  |  |  |  |  |  | (n = 7,343) | | |
|  | Nulliparous women (n = 3,066) | | | |  | Multiparous women without HDP history (n = 4,043) | | | |  | Multiparous women with HDP history (n = 234) | | | |  |
|  | OR | 95%CI | | |  | OR | 95%CI | | |  | OR | 95%CI | | |  |
| Women with low birthweight | 1.16 | 0.54 | – | 2.50 |  | 1.48 | 0.82 | – | 2.67 |  | 5.41 | 1.75 | – | 16.75 |  |
| GDM history and/or  GDM during the most recent pregnancy | 1.00 | 0.36 | – | 2.77 |  | 0.99 | 0.42 | – | 2.35 |  | 1.08 | 0.22 | – | 5.21 |  |
| Age at the follow-up assessment (years) | 1.04 | 1.00 | – | 1.09 |  | 1.09 | 1.05 | – | 1.14 |  | 1.09 | 1.00 | – | 1.19 |  |
| BMI at the follow-up assessment (kg/m^2^) | 1.17 | 1.12 | – | 1.22 |  | 1.17 | 1.12 | – | 1.21 |  | 1.10 | 1.02 | – | 1.19 |  |
| Pulse rate at the follow-up assessment (/min) | 1.06 | 1.04 | – | 1.09 |  | 1.06 | 1.04 | – | 1.09 |  | 1.05 | 1.00 | – | 1.11 |  |
| Smoking at the follow-up assessment | 0.77 | 0.30 | – | 1.96 |  | 1.16 | 0.62 | – | 2.16 |  | 0.57 | 0.13 | – | 2.38 |  |
| Drinking the follow-up assessment | 0.95 | 0.60 | – | 1.50 |  | 1.95 | 1.34 | – | 2.84 |  | 1.61 | 0.72 | – | 3.60 |  |
| Paternal history of chronic hypertension | 1.49 | 0.95 | – | 2.32 |  | 1.52 | 1.02 | – | 2.25 |  | 2.64 | 1.13 | – | 6.15 |  |
| Maternal history of chronic hypertension or HDP | 2.70 | 1.73 | – | 4.21 |  | 1.97 | 1.34 | – | 2.92 |  | 1.46 | 0.63 | – | 3.37 |  |
| HDP during the most recent pregnancy | 5.59 | 3.61 | – | 8.68 |  | 5.67 | 3.76 | – | 8.55 |  | 3.24 | 1.41 | – | 7.44 |  |
| HDP; hypertensive disorders of pregnancy, BMI; body mass index, GDM; gestational diabetes mellitus, OR; odds ratio, CI; confidence interval. | | | | | | | | | | | | | | |  |
