# Supplementary figures and images for: Subsequent high blood pressure and hypertension by hypertensive disorders of pregnancy: the Tohoku Medical Megabank Project Birth and Three-Generation Cohort Study
Source: Hypertens Res. 2024 Oct 11;48(1):68–76. doi: 10.1038/s41440-024-01936-9 (PMC11700841; doi:10.1038/s41440-024-01936-9)

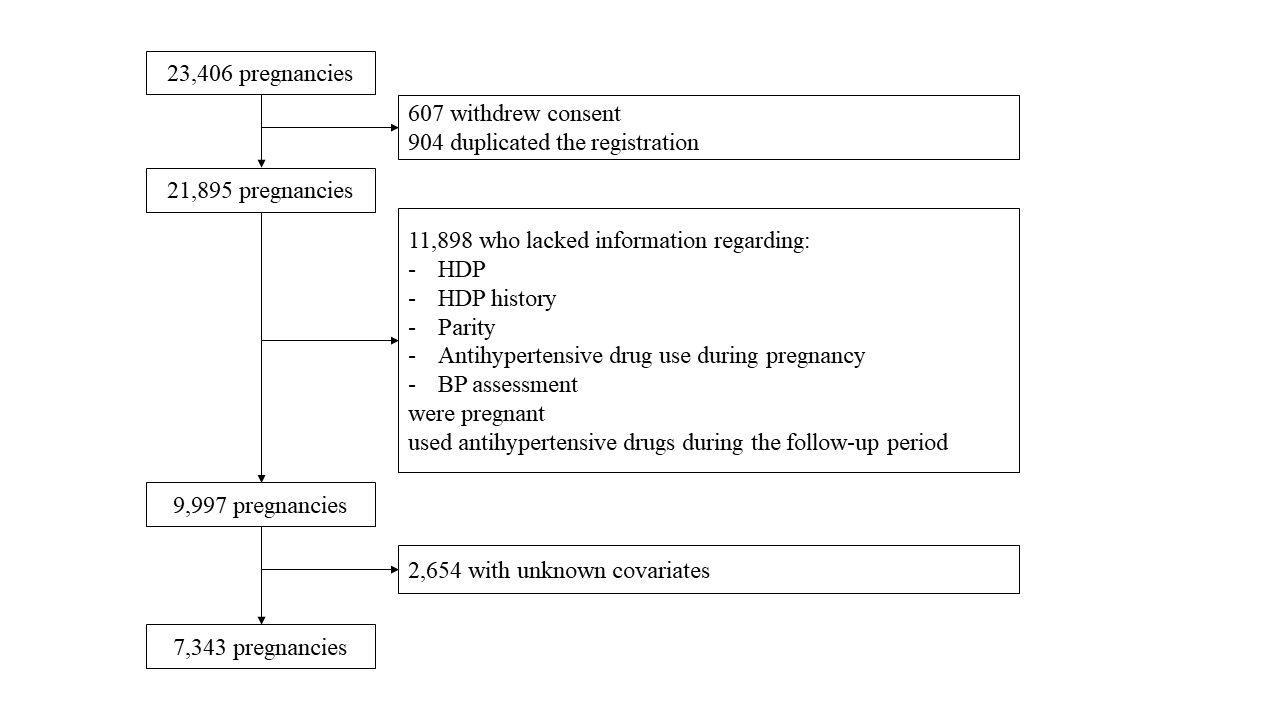

Supplement: Supplementary file 2 — Supplementary Figure 1 [file 41440_2024_1936_MOESM2_ESM.tif]
