# Supplementary material for: Subsequent high blood pressure and hypertension by hypertensive disorders of pregnancy: the Tohoku Medical Megabank Project Birth and Three-Generation Cohort Study
Source: Hypertens Res. 2024 Oct 11;48(1):68–76. doi: 10.1038/s41440-024-01936-9 (PMC11700841; doi:10.1038/s41440-024-01936-9)
Supplement: Supplementary file 3 — Supplementary Figure Legend [file 41440_2024_1936_MOESM3_ESM.docx]

Supplementary Figure 1. Participants selection

It illustrates the participants flow diagram.
